# Molecular Survey of Selected Bacterial Respiratory Pathogens in Polish Wild Boars

**DOI:** 10.3390/pathogens14121196

**Published:** 2025-11-24

**Authors:** Ewelina Czyżewska-Dors, Agnieszka Nowak, Sylwia Zębek, Arkadiusz Dors

**Affiliations:** 1 Department of Internal Diseases and Diagnostics, Poznan University of Life Sciences, Szydłowska 50, 60-637 Poznań, Poland; 2 Department of Bacteriology and Bacterial Animal Diseases, National Veterinary Research Institute, Partyzantów 57, 24-100 Puławy, Poland; 3 Department of Preclinical Sciences and Infectious Diseases, Poznan University of Life Sciences, Wołyńska 35, 60-637 Poznań, Poland

**Keywords:** wild boars, co-infections, bacterial agents, PRDC, Poland

## Abstract

Wild boar (*Sus scrofa*) is a widespread invasive species in Poland and may act as a reservoir for various pathogens, including those associated with the porcine respiratory disease complex (PRDC). As data on bacterial respiratory pathogens in wild boar populations, particularly co-infections, in Poland and other European countries remain limited, the main goal of our study was to examine the frequency of selected bacterial respiratory agents and their co-occurrence in lung samples collected from culled wild boars during hunting. Two hundred and fifty-three lung samples were analysed for the presence of genetic material of *A. pleuropneumoniae*, *M. hyopneumoniae*, *M. hyorhinis*, and *G. parasuis*. In total, 159 out of the 253 (62.8%; 95% CI: 56.6–68.8) wild boars were infected with at least one pathogen. In general, 73 (28.9%; 95% CI: 23.3–34.9) of wild boar lung samples tested positive for *G. parasuis*, 106 (41.9%; 95% CI: 35.7–48.2) were positive for *M. hyopneumoniae*, and 10 (4%; 95% CI: 1.9–7.1) were positive for *M. hyorhinis*. No evidence of *A. pleuropneumoniae* infection was detected in any of the examined lung samples. Infection with a single pathogen was detected in 129 (51%; 95% CI: 44.6–57.3) of sampled wild boars, whereas co-occurrence of two infectious bacterial agents was revealed in 30 animals (11.9%; 95% CI: 8.1–16.5). Among single-pathogen infections, the highest positivity rate was observed for *M. hyopneumoniae* (31.6%; 95% CI: 25.9–37.7), whereas the most frequent co-infection involved *M. hyopneumoniae* and *G. parasuis* (9.1%; 95% CI: 5.8–13.3). This investigation indicated that wild boar in the study area are potential hosts for bacterial agents associated with PRDC. It is worth highlighting that wild boars can contribute to the maintenance and/or dissemination of bacterial pathogens to humans (especially hunters) and domestic animals, and it is essential to maintain active surveillance of these infectious agents.

## 1. Introduction

Wild boars (*Sus scrofa*) are native to many countries worldwide, including Poland. Favourable environmental conditions, ecological plasticity across diverse habitats, and a propensity for continuous reproduction contribute significantly to the expansion of the wild boar population across Europe [1,2]. The wild boar population in Poland is in line with those of Spain, Italy, France, and Germany, resulting in an estimated population density throughout Europe of up to 15 individuals/km^2^ [3].

Wild boars can even be found in cities due to the ease of obtaining food, mainly from unsecured household food waste. Besides causing significant losses due to crop destruction, wild boars can serve as reservoirs of various pathogens, including zoonotic agents such as Hepatitis E virus, *Salmonella* spp., and *Brucella* spp., as well as pathogens shared with domestic pigs, such as *Mycoplasma hyopneumoniae* (*M. hyopneumoniae*) and *Actinobacillus pleuropneumoniae* (*A. pleuropneumoniae*), which can be transmitted to both livestock and humans [1,2,4]. The risk of disease transmission between wild boars and domestic pigs is highest in backyard or outdoor pig production systems, as these systems facilitate direct or indirect contact with wild boars. Currently, outdoor pig production, primarily organic farming in EU countries, accounts for a minor portion of total pig production. Currently, fewer than 2% of pigs in the EU are kept under backyard systems and/or organic production. However, consumer demand for free-range pork, especially organic production, continues to grow [5,6,7,8].

Among the variety of pathogens, wild boars can harbour and potentially transmit porcine respiratory disease complex (PRDC)-related bacterial agents, infection with which in pigs may lead to severe economic consequences [9,10]. PRDC is a complex disease with multiple contributing factors, arising from viral and bacterial infections, as well as environmental and management influences [11]. Among bacterial agents, *Actinobacillus pleuropneumoniae* (*A. pleuropneumoniae*) and *Mycoplasma hyopneumoniae* (*M. hyopneumoniae*) are considered primary pathogens involved in the aetiology of PRDC. *Glaesserella parasuis* (*G. parasuis*) and *Mycoplasma hyorhinis* (*M. hyorhinis*) are opportunistic pathogens frequently detected in co-infections with primary viral and/or bacterial pathogens related to PRDC. The occurrence of co-infection has a significant impact on the clinical presentation of the disease, typically exacerbating the severity of the infection and the extent of lung lesions [12]. Studies conducted in Europe have revealed that pneumonia and pleuritis are the most frequent lung lesions observed at slaughterhouses, with prevalence rates of up to 69% and 48%, respectively [13]. A study conducted by Ferraz et al. [14] in 2020 estimated a loss of $6.55 per animal with lung lesions at slaughter, compared to those without.

Data regarding bacterial respiratory pathogens in the wild boar population, especially co-infection in Poland, as well as in other European countries, are obscure. Thus, the main goal of our study was to investigate the frequency of selected bacterial respiratory agents (mainly those related to PRDC) and co-infections in lung samples collected from wild boars post-mortem during hunts. To evaluate the frequency of infection of *A. pleuropneumoniae*, *M. hyopneumoniae*, *M. hyorhinis* and *G. parasuis* in wild boars in Poland, the PCR assays were conducted.

## 2. Materials and Methods

### 2.1. Sampling

**Figure 1:**
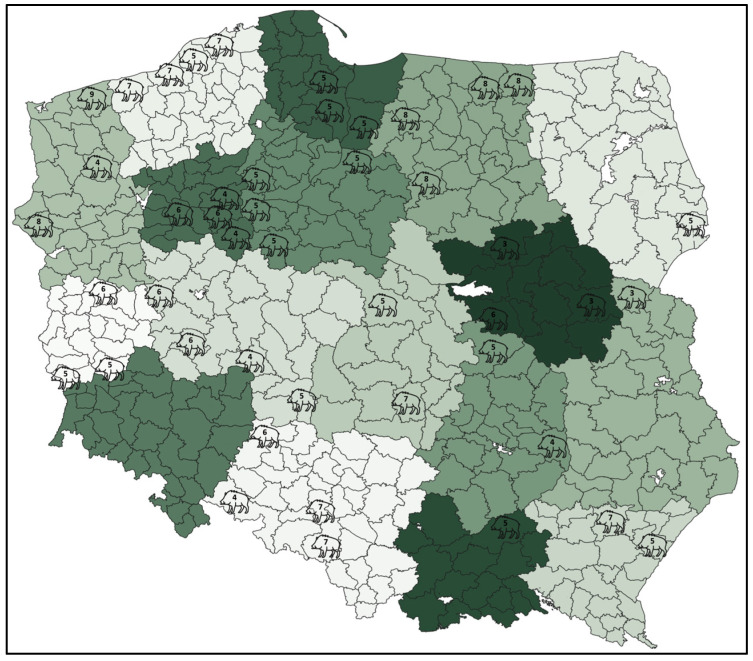
Number of wild boars sampled in each forest inspectorate.

Lung samples were collected during hunting season 2017–2018 and 2018–2019 in 40 forest inspectorates belonging to 16 of 17 Regional Directorates of State Forests (RDSFs) in Poland (Figure 1). All lung samples were collected post-culling during the evisceration process.

Based on body weight, the hunted boars were categorised into three age groups: juveniles (up to 35 kg, less than 1 year), adolescents (36 to 70 kg, between 1 and 2 years), and adults (more than 70 kg, over 2 years)—or classified as animals whose age could not be determined. In total, samples were collected from 253 wild boars, comprising 20 juveniles, 135 adolescents, 87 adults and 11 individuals of undetermined age class. Collected lung samples were sent under cooling conditions to the laboratory and then refrigerated at −20 °C until further analysis.

### 2.2. DNA Extraction and PCR for Pathogen Detection

To obtain genetic material, lung tissue homogenates (50% wt/vol) were prepared in PBS from lung tissue fragments. DNA was extracted from previously prepared samples using the Genomic Mini DNA isolation kit (A&A Biotechnology, Gdańsk, Poland) according to the manufacturer’s recommendations. The obtained DNA was stored at −70 °C for further analysis.

DNA from lung tissue homogenates was used for the detection of bacterial pathogens (*M. hyopneumoniae*, *G. parasuis*, and *M. hyorhinis*) by individual real-time PCR assays, while conventional PCR was used for *A. pleuropneumoniae* detection. All reactions, except for *A. pleuropneumoniae*, were performed using the Master Mix QuantiTect Probe PCR kit (Qiagen, Germantown, MD, USA) according to the manufacturer’s instructions in a final volume of 25 μL, with 20 μM each primer and 10 μM probe. Detection of *A. pleuropneumoniae* DNA was performed according to a previously published procedure [15]. Products obtained in conventional PCR were separated by electrophoresis in a 2% agarose gel. Fluorescence gel images were taken under UV light using EC3 Chemi HR 410 Imaging System (UVP, Upland, CA, USA). The primers used in the present study are presented in Table 1.

### 2.3. Statistical Analysis

The proportion of seropositivity and the corresponding 95% confidence intervals (95% CI) were calculated using the exact Clopper-Pearson method with an online tool (https://statpages.info/confint.html, accessed on 5 March 2025). Differences in the presence of genetic material for different pathogens among different age groups were calculated with the chi-square test or Yates’s chi-square test, with Bonferroni correction for pairwise comparisons. *p*-values < 0.05 were considered statistically significant. Microsoft Excel 2019 (version 2409; Microsoft, Redmond, WA, USA) and the Real Statistics Resource Pack for Excel (Release 9.1.1; Charles Zaiontz, Trento, Italy) were used for data analysis.

## 3. Results

### 3.1. Prevalence of Respiratory Bacterial Pathogens Infection and Co-Infection

**Figure 2:**
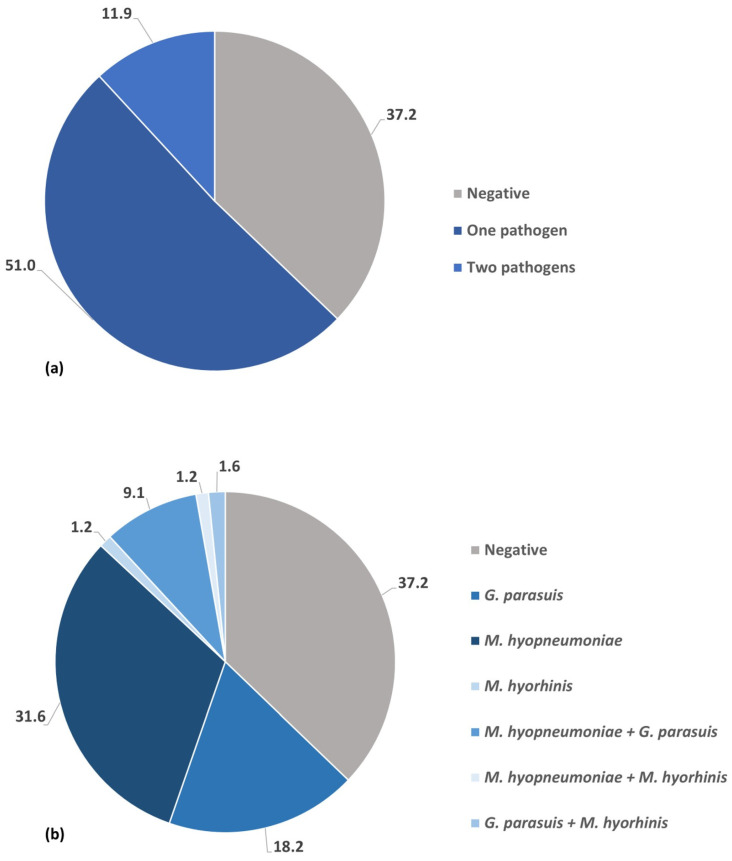
(**a**) Frequency (%) of single-pathogen infection and co-infections. (**b**) Frequency (%) of specific single-pathogen infections and co-infections.

Out of the 253 wild boars, 159 (62.8%; 95% CI: 56.6–68.8) were infected with at least one pathogen. In general, 73 (28.9%; 95% CI: 23.3–34.9) of wild boar lung samples tested positive for *G. parasuis*, 106 (41.9%; 95% CI: 35.7–48.2) were positive for *M. hyopneumoniae*, and 10 (4%; 95% CI: 1.9–7.1) were positive for *M. hyorhinis*. None of the examined lung samples showed evidence of *A. pleuropneumoniae* infection. One hundred and twenty-nine (51%; 95% CI: 44.6–57.3) of the sampled wild boars presented evidence of infection with a single pathogen. Simultaneous occurrence of two infectious bacterial agents was revealed in 30 animals (11.9%; 95% CI: 8.1–16.5) (Figure 2a). Among single-pathogen infection the highest rate of positivity was detected for *M. hyopneumoniae*—31.6% (95% CI: 25.9–37.7), followed by *G. parasuis*—18.2%% (95% CI: 13.6–23.5) and *M. hyorhinis*—1.2%; (95% CI: 0.2–3.4) (Figure 2b). Infection with two different pathogen was detected in 11.9% (95% CI: 8.2–16.5) of tested animals. The most common co-infection was *M. hyopneumoniae* and *G. parasuis*—9.1% (95% CI: 5.8–13.3), while the co-occurrence of *M. hyopneumoniae* and *M. hyorhinis*—1.2% (95% CI: 0.2–3.4) as well as *G. parasuis* and *M. hyorhinis*—1.6% (95% CI: 0.4–4.0) was less frequent (Figure 2b).

### 3.2. Prevalence of Respiratory Bacterial Pathogens Infection and Co-Infection Depending on Age

Regarding the frequency of occurrence of the tested pathogens in the above-mentioned age groups, our study revealed a statistically higher positivity rate of *M. hyopneumoniae* in adolescents compared to adult wild boars, and between juveniles and the adolescent age group in terms of *M. hyopneumoniae* and *G. parasuis* co-infection (*p* < 0.05) (Table 2).

## 4. Discussion

The present study reports the prevalence of *M. hyopneumoniae*, *A. pleuropneumoniae*, *G. parasuis*, and *M. hyorhinis*, as well as the co-occurrence of these bacterial agents in wild boars in Poland. To the best of the authors’ knowledge, this is the first study to comprehensively describe the prevalence of various bacterial pathogens in wild boar lung samples in European countries. Due to the potential transmission of infection from wild boars to pigs, monitoring wild boars for pathogens dangerous to pigs, especially those of economic and zoonotic impact, seems highly justified [10].

The overall results presented here demonstrated that more than half of the animals carried genetic material of only a single pathogen, while only 11.5% of wild boars were co-infected with two different pathogens. Notably, 37.2% of them did not harbour any of the bacterial pathogens surveyed.

*A. pleuropneumoniae* is one of the major bacterial porcine respiratory tract pathogens causing pleuropneumonia outbreaks worldwide. Although it is a pathogen capable of causing infection on its own, it is often isolated together with other bacteria and/or viruses [15]. A meta-analysis of porcine respiratory tract coinfections data indicates that secondary infection with *A. pleuropneumoniae* can aggravate swine influenza and *M. hyopneumoniae*-related disease. Surviving the *A. pleuropneumoniae* infection leads to persistent bacterial carriage in the tonsils or lungs sequesters [19]. In the present study, none of the tested lung samples were positive for *A. pleuropneumoniae*. This finding aligns with an Australian study that examined lung samples from feral pigs and demonstrated their freedom from *A. pleuropneumoniae* DNA using PCR [20]. Similarly, a very low prevalence of *A. pleuropneumoniae* DNA was also observed in Brazil, where 1 out of 79 samples tested positive [9]. On the other hand, a German study detected *A. pleuropneumoniae* in 34.5% of tonsil samples and 6.4% of lung samples, suggesting that wild boars may be carriers of the aforementioned pathogen [21]. Also, a study conducted in Hungary revealed that 14.7% of examined wild boars harbour *A. pleuropneumoniae* in their tonsil [22]. Considering that the prevalence of *A. pleuropneumoniae* DNA in commercial pigs is considerably higher than in wild boars, approaching 50% positive lung samples, wild boar populations might be at risk of infection from domestic pig herds [23]. It is worth noting that previous studies have indicated that the frequency of *A. pleuropneumoniae* DNA increases with the age of the tested wild boars [21,24]. In our research, no sample was positive, so it was not possible to perform such an analysis.

*Mycoplasma hyopneumoniae*, the etiologic agent of enzootic pneumonia (EP), a chronic respiratory disease with high morbidity and low mortality, is another major pathogen involved in PRDC in pigs. The role of *M. hyopneumoniae* as a facilitator of secondary bacterial infections, particularly *A. pleuropneumoniae*, *P. multocida*, and *M. hyorhinis*, is well established [25]. Based on previous serological and molecular pieces of evidence, it is known that *M. hyopneumoniae* is able to infect the wild boar. Molecular study conducted in Spain revealed that in 17 out of 85 (20%) nasal swabs and in 12 out of 156 (8%) lung samples, DNA of *M. hyopneumoniae* was detected by nested PCR [26]. Moreover, in all the studied wild boars, enzootic pneumonia (EP)-like gross lesions were observed. Furthermore, the presence of EP-like microscopic lung lesions was observed in 29% of the tested animals, indicating that wild boars could have a subclinical form of the disease [26]. In our study, genetic material of *M. hyopneumoniae* was detected in more than one-third of the tested samples, which is in line with an Italian study in which *M. hyopneumoniae* DNA was detected in 45% of the tested lung samples [27]. According to Chiari et al. [27], discrepancies between studies resulted from sampling groups of animals with a lower probability of *M. hyopneumoniae* shedding. The author mentioned above revealed that lung samples from juvenile wild boars showed higher lung scores of gross lung lesions compatible with EP than those from subadults and adults, with a positive association with the *M. hyopneumoniae* PCR-positive status [27]. In our study, the frequency of detection was significantly higher in adolescent wild boars compared to adults. It is worth noting that in Brazilian wild boars, no genetic material of *M. hyopneumoniae* was detected in any of the examined lung samples. According to the authors, this finding may suggest a reduced level of contact between wild boars and domestic pigs in the studied region of Brazil [9]. On the other hand, another study conducted in the Brasilia region, Parana, detected *M. hyopneumoniae* in 19 of 25 (76%) lung samples with gross lesion characteristic for EP using the immunohistochemistry (IHC) technique [28]. The differences may result from the use of other methods for *M. hyopneumoniae* detection and the status of the gross lung lesion score in the samples.

*Glaesserella parasuis* is the specific pathogenic cause of Glässer’s disease in swine, which results in polyserositis, including pleuritis, peritonitis, and arthritis [29]. The bacterium is an early coloniser of piglets and is commonly isolated from the upper respiratory tract of healthy individuals; however, its detection in the lower respiratory tract is associated with pneumonia [30].

It has been previously confirmed that wild boars can serve as a reservoir for *G. parasuis*, and domestic pigs are at risk of infection. Reiner et al. [30] identified *G. parasuis* DNA in 69.1% of tonsil samples and 40.4% of lung samples, with an overall prevalence of *G. parasuis* in wild boars in Germany reaching 74.2%. However, no individuals exhibited visible lesions indicative of Glässer’s disease. The same authors also investigated whether sex, age, or body weight might influence the infection status, and they concluded that it had no effect, which is consistent with our results. In Spain, evidence of infections with *G. parasuis* in lung tissue was not detected [24]. However, a previous study from Spain isolated *G. parasuis* from nasal swabs of hunted wild boars [31]. Moreover, in Spain, a fatal case of wild boar mortality due to Glässer’s disease caused by *G. parasuis* has been reported [32]. The pathogenic potential and disease development vary according to different strains and serovars, individual host resistance, age, colostral protection, and herd health status and origin [32].

*Mycoplasma hyorhinis* is a commensal bacterium often found in the porcine respiratory tract and tonsil [33]. It has been considered a possible primary pathogen of several porcine diseases, including arthritis, conjunctivitis, otitis media, eustachitis, meningitis, and pneumonia [34,35]. The role of *M. hyorhinis* in the development of pneumonia in pigs remains unclear, although some authors suggest that this species can induce mild pneumonia even in the absence of *M. hyopneumoniae* [36,37,38]. Studies conducted in Switzerland and Germany detected *M. hyorhinis* DNA in 10% and 18.5% of samples with lesions characteristic of EP, respectively [35]. However, an in vivo experimental study conducted by Fourour et al. [39] did not confirm the ability of *M. hyorhinis* to induce lung lesions in SPF pigs independently. Inoculation with *M. hyorhinis* alone led to the detection of the pathogen in serous membranes (polyserositis) but not in bronchi. However, co-infection of SPF pigs first with *M. hyopneumoniae* and subsequently with *M. hyorhinis* resulted in the detection of *M. hyorhinis* in the bronchi and *M. hyopneumoniae* in the serous membranes [39]. Regarding wild boars, the literature does not contain any information on the prevalence of *M. hyorhinis* in this species. The present study is the first to confirm the occurrence of *M. hyorhinis* DNA in wild boars. It is worth emphasising that *M. hyorhinis* has been identified as a causative agent of severe pneumonia in adult patients. The likely source of infection was pork meat, as the patient had no contact with live pigs. This report suggests that *M. hyorhinis* may have a zoonotic potential, highlighting the risk associated with contact with infected pigs and/or wild boars [40].

Co-infections were also assessed in the present study. Of the tested animals, 11.5% were shown to be co-infected. Among the combination of co-infection, *M. hyopneumoniae* and *G. parasuis* were frequently detected (8.7%). Simultaneous infection of the pig respiratory tract by different pathogens is detected worldwide at a very high rate [25,41,42]. Regarding wild boars, most studies were focused on detecting the presence of individual pathogens in the respiratory tract, but not co-infections. Studies describing co-infections using molecular methods are scarce.

It is well known that, in most cases, respiratory tract co-infection leads to a more severe clinical outcome of the disease compared to a single infection [12,42]. A previous study conducted in Brazil attempted to determine the co-occurrence of pathogens involved in PRDC in wild boars using molecular methods [9]. Among bacterial pathogens, the presence of *A. pleuropneumoniae*, *G. parasuis*, *P. multocida*, and *M. hyopneumoniae* was examined in the aforementioned study using DNA in lung samples. In addition, the genetic material of porcine circovirus type 2 (PCV2), *Torque teno sus virus 1a* (TTSuV1a) and 1b (TTSuV1b) were also tested in lymph nodes. Similarly to our result, 11.3% of tested animals presented co-infection. However, TTSuV1a was the most common pathogen present in the animals with co-infections, being present in all combinations. Co-infection with two different bacterial agents was not detected in any of the tested samples. Generally, bacteria-related pathogens were detected in only 8.8% of the samples, whereas viral genomes were detected in 56.3% of the tested samples. The authors also examined the likelihood of infection in relation to age, and the evaluated variables were not associated with any pathogen [9].

In our study, statistical differences in the prevalence of *M. hyopneumoniae* and *G. parasuis* co-infection were detected between the juveniles and adolescents age groups. In Spain, co-infections between different pathogens in wild boars have also been studied, specifically in terms of their impact on bovine tuberculosis severity [22]. Results of the mentioned study revealed that contact with PCV2, Aujeszky’s disease virus (ADV) and infection by *Metastrongylus* spp. were positively correlated with tuberculosis severity, but swine influenza A virus (swIAV), porcine reproductive and respiratory syndrome virus (PRRSV), *M. hyopneumoniae*, *A. pleuropneumoniae*, and *G. parasuis* were not factors related to tuberculosis severity [24].

## 5. Conclusions

In conclusion, bacterial infectious agents related to PRDC, such as *M. hyopneumoniae*, *G. parasuis*, and *M. hyorhinis* (the first confirmation of its presence in wild boars), have been demonstrated to infect the Polish wild boar population and may represent a health concern related to infectious diseases in pigs. It is worth emphasising that no genetic material of *A. pleuropneumoniae* was detected in any of the animals, indicating that there is no risk of transmission of this bacterium from wild boars to pigs. Although contact between domestic pigs and wild boars is unlikely in the study area due to the dominance of indoor production systems in domestic pigs, it is essential to maintain active surveillance for infectious pathogens, including not only bacterial but also viral pathogens associated with PRDC in the wild boar population.

## Figures and Tables

**Table 1:** Oligonucleotide primer and probe sequences used in the study.

Pathogens	Target (Gene)	Oligonucleotide Sequences (5′-3′)	Reference
*A. pleuropneumoniae*	*apxIVA*	apxIVA1-F: TGGCACTGACGGTGATGA apxIVA1-R: GGCCATCGACTCAACCAT	[15]
*G. parasuis*	*infB*	CTinfF1: CGACTTACTTGAAGCCATTCTTCTT CTinfR1: CCGCTTGCCATACCCTCTT CTinfP: FAM-ATCGGAAGTATTAGAATTAAGTGC-TAMRA	[16]
*M. hyopneumoniae*	*p102*	P102f: GTCAAAGTCAAAGTCAGCAAAC P102r: AGCTGTTCAAATGCTTGTCC P102 probe: Cy5-ACCAGTTTCCACTTCATCGCCTCA-BHQ2	[17]
*M. hyorhinis*	*p37*	Mhr-p37-RT-F: TATCTCATTGACCTTGACTAAC Mhr-p37-RT-R: ATTTTCGCCAATAGCATTTG Mhr-p37-Probe: FAM-CATCCTCTTGCTTGACTACTCCTG-BHQ1	[18]

**Table 2:** Occurrence of genetic material against analysed pathogens detected in wild boar depending on age category (Juveniles (*n* = 20); Adolescents (*n* = 135); Adults (*n* = 87)).

Pathogens	Juveniles		Adolescents		Adults	
	Number (%) of Positive	95% CI	Number (%) of Positive	95% CI	Number (%) of Positive	95% CI
***G. parasuis***	7 (35.0%)	18.1–56.7	20 (14.8%)	9.8–21.8	18 (20.7%)	13.5–30.4
***M. hyopneumoniae***	5 (25.0%)	11.2–46.9	55 (40.7% ^a^)	32.8–49.2	17 (18.5% ^b^)	12.6–29.0
***M. hyorhinis***	0 (0%)	0–16.1	2 (1.5%)	0.4–5.2	1 (1.1%)	0.2–6.6
***M. hyopneumoniae* + *G. parasuis***	5 (25.0% ^a^)	11.2–46.9	8 (5.9% ^b^)	3.0–11.3	7 (8.1%)	4.0–15.7
***M. hyopneumoniae* + *M. hyorhinis***	0 (0%)	0–16.1	2 (1.5%)	0.4–5.2	1 (1.1%)	0.2–6.6
***G. parasuis* + *M. hyorhinis***	0 (0%)	0–16.1	2 (1.5%)	0.4–5.2	2 (2.3%)	0.6–8.0

^a^, ^b^—different letters represent a statistically significant difference between the analysed groups; 95% CI—95% confidence interval.

## Data Availability

All necessary data are contained within the article.

## Abbreviations

The following abbreviations are used in this manuscript:

ADV	Aujeszky’s disease virus
EP	enzootic pneumonia
IHC	immunohistochemistry
PCV2	porcine circovirus type 2
PRDC	porcine respiratory disease complex
PRRSV	porcine reproductive and respiratory syndrome virus
swIAV	swine influenza A virus
TTSuV1a	*Torque teno sus virus 1a*
TTSuV1b	*Torque teno sus virus 1b*
